# Thrombosed Mechanical Aortic Valve Treated with Low-Dose Ultraslow Alteplase Infusion

**DOI:** 10.3390/medicines12010003

**Published:** 2025-02-02

**Authors:** Nicholas Pavlatos, Pawan Daga, Aangi Shah, Muhammad Khan, Jishanth Mattumpuram

**Affiliations:** 1 Department of Internal Medicine, University of Louisville School of Medicine, Louisville, KY 40202, USA; 2 Division of Cardiology, University of Louisville School of Medicine, Louisville, KY 40202, USAjishanth.mattumpuram@louisville.edu (J.M.)

**Keywords:** prosthetic valve, echocardiography, fluoroscopy, anticoagulation

## Abstract

**Background**: Prosthetic valve thrombosis is a rare but serious complication of mechanical valve replacement. Traditionally, prosthetic valve thrombosis has been managed by surgical intervention; however, there is increasing data to support the use of thrombolytics. **Methods**: We present a case of a 74-year-old female with a history of rheumatic fever and subsequent mechanical aortic valve replacement on warfarin who presented to the emergency department with disequilibrium and chest pain. **Results**: She was found to have a subtherapeutic international normalized ratio and thrombosed mechanical aortic valve seen on transthoracic echocardiography, transesophageal echocardiography, and fluoroscopy. **Conclusions:** She was treated with a low-dose ultraslow alteplase infusion of 25 mg of alteplase administered over 25 h. Post-infusion transthoracic echocardiography immediately following infusion and four months later confirmed resolution of thrombosis.

## 1. Introduction

Prosthetic valve thrombosis (PVT) is typically an acute event that results in rapid valve dysfunction due to abnormal or absent motion of the valve leaflets. The risk of PVT is higher in mechanical than bioprosthetic valves, with an annual rate of mechanical valve thrombosis ranging between 0.1 and 5.7%. Although subtherapeutic anticoagulation is the primary contributing factor for PVT, higher rates are seen in the mitral versus aortic valves, right-sided versus left-sided prosthetic valves, presence of atrial fibrillation, certain valve types, and early in the postoperative period [1,2,3].

PVT may be found incidentally on echocardiogram or present with thromboembolism, presyncope, syncope, sudden death, or signs and symptoms of heart failure such as progressive dyspnea, cough, edema, fatigue, and cardiogenic shock [4].

Patients with suspected PVT should undergo transthoracic echocardiogram (TTE) to determine whether valve obstruction is present. The American Heart Association (AHA)/American College of Cardiology (ACC) guidelines define prosthetic aortic valve obstruction as an increase in mean transvalvular gradient by >50% or by >10 mmHg over baseline [5]. Once initial evaluation with TTE suggests PVT, additional evaluation with transesophageal echocardiography (TEE), multidetector computed tomography, and fluoroscopy can aid in confirmation of diagnosis and help guide treatment.

Upon the diagnosis of PVT, a heart valve team should make an individualized assessment before choosing between medical management with systemic fibrinolysis or surgical management with valve replacement or thrombectomy. AHA/ACC guidelines favor systemic fibrinolysis in patients when no surgical expertise is available, at high surgical risk, with the presence of a mass consistent with thrombus, at the first episode of valve thrombosis, with New York Heart Association Class I, III, or III, with no left atrial thrombus, with no need for other cardiac surgery such as coronary artery bypass graft or other valve disease, and with no contraindication to fibrinolysis [5]. Currently, the European Society of Cardiology makes no recommendations regarding the use of fibrinolytics in PVT beyond its use for cases in which surgical risk is too high or not available [6]. Although fibrinolytic therapy has established itself as a first line treatment in the management of PVT, there are currently no guidelines regarding choice of agent, dose, route, or duration of administration.

## 2. Patient Case

Our patient was a 74-year-old female with a history of paroxysmal atrial fibrillation, hypertension, and rheumatic heart disease status post-mechanical aortic valve replacement, who presented to the emergency department with a one-week history of imbalance and chest pain. The patient had a St. Jude bileaflet mechanical aortic valve placed in 1995 and had been on warfarin with an international normalized ratio (INR) goal of 2–3. She noticed that over the previous week she had been having trouble ambulating, stating that she had been favoring one side and tripping over her own feet. Additionally, she endorsed chest pain, which was described as constant, midsternal, and exertional. On physical exam, the patient had slurred speech, left sided facial droop, and pain on palpation of her anterior chest wall.

Workup in the emergency department was significant for high sensitivity troponin of 155 ng/L, INR of 1.5, hemoglobin of 11.1 g/dL with occasional schistocytes on peripheral smear, and a normal computed tomography of her head. EKG on arrival showed the patient was in normal sinus rhythm, with a right bundle branch block and left ventricular hypertrophy consistent with previous electrocardiograms.

**Figure 1:**
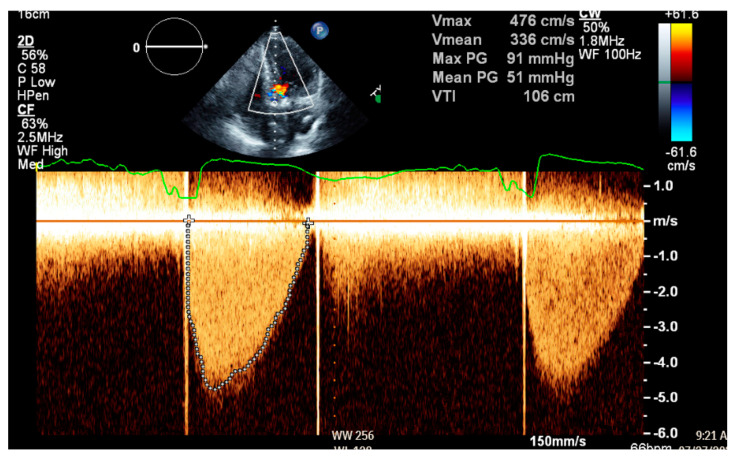
Transthoracic echocardiography Doppler prior to ultraslow low-dose alteplase infusion showing a mean aortic transvalvular gradient of 51 mmHg and aortic valve peak velocity of 494 cm/s, consistent with severe aortic stenosis.

**Figure 2:**
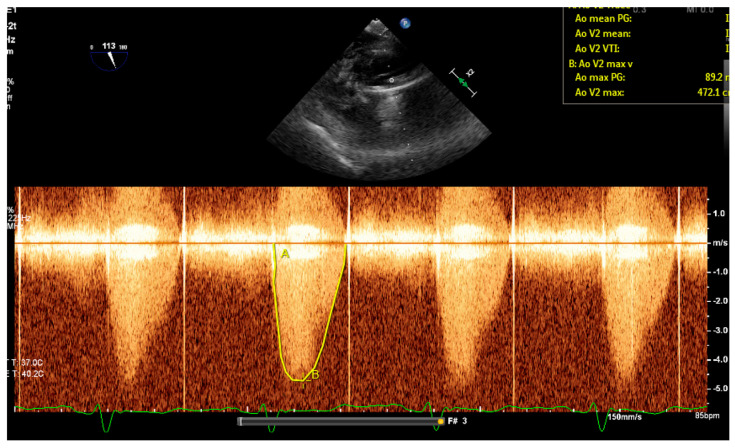
Transesophageal echocardiography Doppler prior to ultraslow low-dose alteplase infusion showing a mean aortic transvalvular gradient of 52 mmHg and aortic valve peak velocity of 472 cm/s, consistent with severe aortic stenosis.

Given the patient’s subtherapeutic INR, she was started on a heparin drip. A TTE was obtained due to concern of obstruction of her prosthetic valve, revealing normal left ventricular function with an ejection fraction of 67%. The mechanical aortic valve was poorly visualized; however, severe aortic stenosis was suspected, as she had a mean aortic transvalvular gradient of 51 mmHg and aortic valve peak velocity of 494 cm/s (Figure 1). Doppler contour was rounded and late peaking, with an acceleration time of 110 ms and peak velocity > 5 m/s. These values were significantly increased from her last documented TTE, in which she had a mean aortic transvalvular gradient of 9 mmHg and aortic valve peak velocity of 219 cm/s. Given poor visualization of the mechanical aortic valve, TEE was performed and confirmed severe aortic stenosis, with a mean aortic transvalvular gradient of 52 mmHg and aortic valve peak velocity of 472 cm/s (Figure 2). Better valve visualization through TEE was able to show incomplete movement of one leaflet of the mechanical aortic valve. Further evaluation with fluoroscopy revealed a bicuspid mechanical aortic valve with free movement of only one leaflet, while the other leaflet appeared immobile. The cause of her elevated aortic valve pressure gradient was believed to be a thrombosed mechanical valve secondary to subtherapeutic INR.

**Figure 3:**
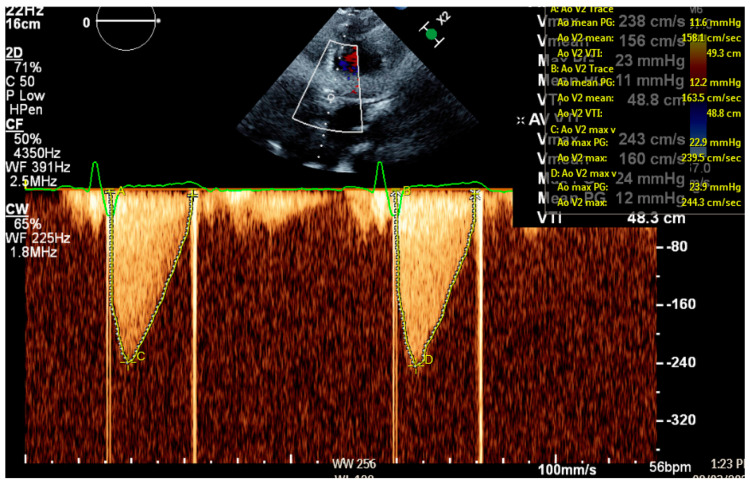
Transthoracic echocardiography following ultraslow low-dose alteplase infusion showing a mean aortic transvalvular gradient of 12 mmHg and aortic valve peak velocity of 283 cm/s, indicating resolution of prosthetic valve thrombosis.

**Figure 4:**
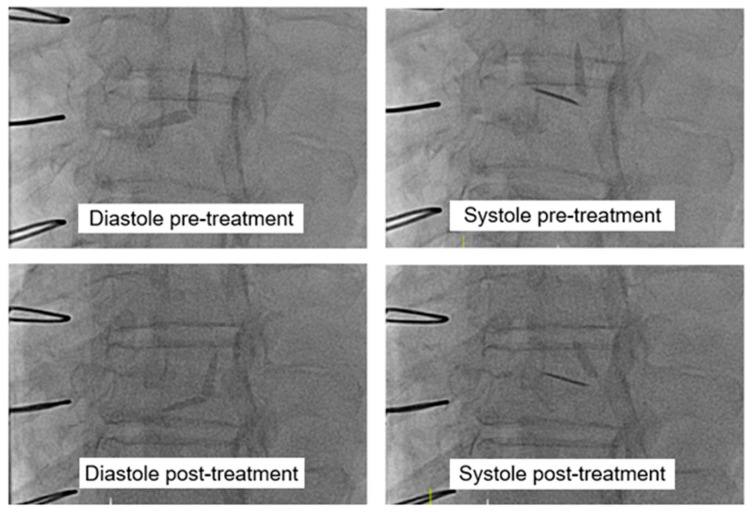
Fluoroscopy of mechanical aortic valve in both systole and diastole pre- and post-treatment with ultraslow low-dose alteplase infusion, revealing improved valve leaflet motion.

The heart valve team met with the patient and agreed she was a candidate for fibrinolytic therapy due to AHA/ACC guidelines and given that this was her first occurrence of PVT, her low New York Heart Association Class, and lack of indications for other cardiac-related surgeries. The patient also expressed her preference to not undergo surgical intervention. Once her INR was below 2.5, she would be started on low-dose ultraslow alteplase infusion of alteplase 25 mg over 25 h followed by heparin drip for six hours. She would be started on therapeutic enoxaparin prior to bridging to warfarin. Post-infusion TTE demonstrated resolution of her aortic stenosis, with a mean aortic transvalvular gradient of 12 mmHg and aortic valve peak velocity of 283 cm/s (Figure 3). The Doppler contour was triangular and early peaking, with an acceleration time of 80 ms and peak velocity < 3 m/s. Repeat fluoroscopy confirmed free movement of both valve leaflets, with no evidence of residual thrombosis (Figure 4). The patient declined coronary angiography as she did not want any invasive procedures despite the risk of embolization resulting in myocardial infarct.

She was discharged on warfarin 5 mg daily with a goal INR of 3.0–3.5. Four months later, the patient remained symptom-free, and repeated TTE at that time showed a mean aortic transvalvular gradient of 16 mmHg, consistent with good valve function.

## 3. Discussion

Multiple small studies comparing the safety and efficacy of fibrinolytic therapy and surgery in the management of PVT have been published, with mixed results. Two meta-analyses have been conducted comparing each intervention; however, they too had conflicting findings. Castilho et al. looked at 48 studies with 2302 participants suffering from PVT and found nearly identical rates of success with fibrinolytic therapy at 80.7% (95% CI, 75.6–85.0) and surgery at 81.9% (95% CI, 77.2–85.8). Significantly less mortality was associated with fibrinolytic therapy at 6.6% (95% CI, 4.8–8.9) than surgical intervention at 18.1% (95% CI, 14.6–22.1). However, other clinically relevant findings favoring surgery over fibrinolytic therapy, respectively, were embolic events (4.6% vs. 12.8%), stroke (4.3% vs. 5.6%), and bleeding (4.6% vs. 6.8%). Recurrence of PVT was not studied [7]. In contrast, Karthikeyan et al. reviewed 53 studies with 690 participants and found no statistically significant difference between surgery and fibrinolytic therapy when comparing success (86.5 vs. 69.7%, OR 2.53, 95% CI, 0.94–6.78, *p* = 0.066) or mortality (13.5 vs. 9%, OR 1.95, 95% CI 0.63–5.98, *p* = 0.244). They did, however, confirm that fibrinolytic therapy was associated with significantly more embolic events, including stroke (16.0% vs. 1.6%) and bleeding (5.0% vs. 1.4%). Importantly, they found that fibrinolytic therapy was significantly more associated with PVT recurrence (25.4% vs. 7.1%) [8]. Between these two meta-analyses, there was only one study that included a group in which low-dose slow infusion fibrinolytic therapy of varying amounts of streptokinase and alteplase over the course of six hours was used [9]. Neither had any data on low-dose ultraslow infusion fibrinolytic therapy, which is now considered standard practice.

There have been multiple studies comparing the dose and length of administration of fibrinolytics in the management of PVT. One study looked at five different groups: streptokinase 1.5 million units over 3 h (Group I), streptokinase 1.5 million units over 24 h (Group II), t-PA 90 mg over 5 h after 10 mg bolus (Group III), t-PA 50 mg over 6 h (Group IV), and t-PA 25 mg over 6 h (Group V). All patients received a TEE within an hour after completion of fibrinolytic therapy; if the obstruction was not resolved, they underwent the same fibrinolytic treatment regimen. The overall success rate was 83.2% and did not differ significantly between each group. There was a significant difference in the complication rate between groups, as Groups I through IV experienced significantly higher complication rates (37.5%, 24.4%, 33.3%, and 29.6%, respectively; *p* > 0.05 for each comparison), whereas Group V had a complication rate of 10.5%, *p* < 0.05 for each. A follow up study administered a low-dose ultraslow alteplase infusion of 25 mg over 25 h to patients with both non-obstructive and obstructive PVT in 114 patients. They found an overall success rate of 90%, with a relatively low complication rate of 6.7%. NYHA Class IV status, presence of atrial fibrillation, smaller valve area, and larger thrombus area were all associated with lower likelihood of success [10].

The AHA/ACC guidelines make no recommendations regarding choice of fibrinolytic or duration of administration [5]. Overall, data comparing fibrinolytic treatment options are lacking in that most studies are conducted at a single center with a limited sample size. The majority of published studies have used alteplase as the fibrinolytic of choice; however, there have been reports of successful treatment of PVT with tenecteplase, reteplase, and streptokinase [11,12,13].

## 4. Conclusions

Choice of management of PVT is difficult in that there is little data comparing systemic fibrinolysis and surgical options. Cardiac surgery is not always available, and surgical mortality remains high despite advances in techniques and perioperative care. Although systemic fibrinolysis has established itself as a treatment option in PVT, recommendations are lacking within the guidelines regarding agent of choice, dose, length of administration, proper monitoring, or follow-up. Recent data suggest that lower doses of fibrinolytics over a prolonged course result in better outcomes. Our case demonstrates that a low-dose ultraslow alteplase infusion can be both safe and effective in the management of mechanical PVT. More research needs to be conducted comparing surgery and low-dose fibrinolytics over a longer period.

## Data Availability

The original contributions presented in the study are included in the article; further inquiries can be directed to the corresponding author.
